# The Role of Identity Formation in Explaining Dynamic Risk Factors Among Incarcerated Emerging Adults

**DOI:** 10.1177/0306624X251329030

**Published:** 2025-03-29

**Authors:** Virginija Klimukiene, Alfredas Laurinavicius, Sandra Bagdonaite, Gintautas Sakalauskas

**Affiliations:** 1 Vilnius University, Lithuania; 2 Independant Researcher

**Keywords:** dynamic risk factors, emerging adults, identity formation, incarceration, offender rehabilitation, correctional treatment, vulnerabilities

## Abstract

Emerging adulthood is increasingly acknowledged as a discrete developmental stage with its own specific challenges. Identity formation is a major development task during emerging adulthood, yet its relationship to dynamic risk factors remains unclear. Therefore, this study aimed to investigate the association between identity diffusion and dynamic risk factors in a sample of young, incarcerated adults. The study involved 99 males between ages of 18 and 23, serving custodial sentences in four Lithuanian prisons. Zero-order correlations showed significant relationships between the length of the current sentence and psychopathology, and the number of previous sentencing occasions and dynamic risk factors. The results of hierarchical regression analysis revealed that identity diffusion explained dynamic risk factors above and beyond criminal history, protective factors, and psychopathology. These findings support a developmentally informed approach to correctional treatment, suggesting that interventions targeting identity diffusion could be suitable and beneficial for young, incarcerated adults. Limitations and future directions are discussed.

Emerging adulthood constitutes a discrete developmental phase, requiring special attention within the criminal justice system (Fountain et al., 2021). Two divergent lines of enquiry are suggestive in this regard. First, youth offenders present with the highest rates of recidivism when compared with the other age groups (Bonta & Andrews, 2017). Second, extensive research on brain development has shown that adolescent brains continue to develop until at least the mid-twenties (Casey et al., 2019), indicating that young adults, with some individual variability, have not fully reached their psychosocial maturity (Monahan et al., 2013). Additionally, most youth desist from crime as they mature (Mendel, 2022). This conceptualizes emerging adulthood as a transition period with a higher risk of criminal activity and susceptibility to external influences. At the same time, this transitional phase offers an exceptional opportunity for early intervention to encourage desistance from crime.

To date, the absence of clear guidelines regarding the appropriate processing of young adults in the criminal justice system remains an unresolved issue. Advocates for a developmentally informed approach encourage reviewing policies associated with young offenders and revising them to reflect their developmental phase (Cauffman et al., 2015). Furthermore, they argue that rehabilitation programs and risk assessments should be tailored to their developmental needs (Farrington et al., 2017). A developmentally sensitive approach seems to be even more necessary, given a range of negative outcomes associated with incarceration at a young age (Cunha et al., 2023; Mendel, 2022).

## Identity Formation

Psychosocial maturity involves the development of a sense of identity, a developmental milestone faced by emerging adults (Kroger et al., 2009). Erikson (1968) defined identity as a fundamental organizing principle that fosters a sense of internal consistency within the self and in the interpersonal milieu (“self-sameness”), while also providing a framework for distinguishing between oneself and others, thereby enabling autonomous functioning (“uniqueness”). From the continuity model perspective, identity achievement and diffusion could be viewed as opposite ends of the spectrum. Identity achievement was found to be associated with more fulfilling interpersonal relationships across settings, more positive self-esteem and better-defined life goals in adolescents and young adults (Foelsch et al., 2008). In contrast, identity diffusion is generally considered to be at the core of psychopathology (Clarkin et al., 2006). According to Kernberg (1989), a person with identity diffusion holds contradictory perceptions about themselves and others, may suffer from chronic feelings of emptiness, may demonstrate contradictory behavior and superficiality, and may present with poor anxiety tolerance and impulse control. Similarly, Marcia (2006) proposed that identity diffusion is generally related to a lack of a coherent life narrative or a sense of continuity over time. There is some empirical research in support of this (Branje et al., 2021).

In terms of associations between identity formation and psychopathology, empirical research to date, including systematic reviews and meta-analyses, showed that identity formation is associated with internalizing problems (Lillevoll et al., 2013), externalizing problems (Crocetti et al., 2013; Schwartz et al., 2010), and personality pathology (Westen et al., 2011). Furthermore, there is a paucity of research studying identity formation within criminal population. Studies have shown that offenders engage in similar identity formation processes to non-offenders (Gavel & Mandracchia, 2016), and that incarceration has a negative impact on identity formation among incarcerated juveniles (Peacock & Theron, 2007). Also, identity diffusion leads to a lack of impulse control, potentially contributing to reactive aggression (Dammann et al., 2011). More recently, Dent and Ward (2023) acknowledged that incarceration could result in an impaired ability to form a coherent and meaningful identity, thus calling for more research to understand identity-related needs that psychological interventions could address in correctional settings.

## Dynamic Risk Factors

The Risk-Need-Responsivity (RNR) model (Bonta & Andrews, 2017) guides risk assessment as well as offender rehabilitation in correctional settings. Within this model, effective rehabilitation targets criminogenic needs (i.e., dynamic risk factors), which, when addressed, are considered to correspond with changes in the likelihood of reoffending. The main dynamic risk factors for young criminal offenders include, but are not limited to, the following: relationships with antisocial peers, family or other main support people, antisocial attitudes, instability and underperformance at school or work, involvement in unstructured leisure activities, substance use, poor impulse control, and emotional dysregulation (Viljoen et al., 2014). Dynamic risk factors are useful for predicting recidivism and assessing the risk state (Douglas & Skeem, 2006). They complement static risk factors, such as a history of offending (Ward, 2016), which determine risk status (Douglas & Skeem, 2006). Studies have shown that a higher level of static risk is associated with a greater likelihood of re-offending and the presence of more dynamic risk factors (Fries et al., 2013; Kurlychek et al., 2006).

The assessment of dynamic risk factors is fundamental for correctional treatment planning. However, some scholars argue that dynamic risk factors are empirically derived constructs and should not be regarded as potential causes of criminal behavior (Beech & Ward, 2004; Ward, 2016). Heffernan and Ward (2017) proposed that these risk/need factors were more reflective of outcomes of underlying psychological issues like insecure attachment, abuse, or past traumatic events. For this reason, they believed that more attention needed to be paid to alternative theories to fully understand offending behavior. Given the correlational, rather than causal, link between dynamic risk factors and criminal behavior, some authors (e.g., Viljoen, Cruise, et al., 2012) suggested referring to them as vulnerabilities. In the current paper the terms “vulnerability” and “dynamic risk factor” will be used interchangeably as suggested by other researchers (e.g., Bui & Deakin, 2021).

The etiological model of risk proposed by Beech and Ward (2004), while primarily focused on sexual offending, provides core principles that are also applicable to other forms of criminal behavior (Brouillette-Alarie & Proulx, 2019). In this model, vulnerabilities consist of both static (e.g., history of offending) and stable dynamic risk factors (e.g., attitudes, general self-regulation problems, interpersonal relationships) and are seen as rooted in developmental issues. When these vulnerabilities interact with acute dynamic risk factors such as emotional states (e.g., anger or excitement) and contextual/situational events (e.g., relationship conflict, presence of antisocial peers), the likelihood of offending behavior increases. In this context, identity diffusion can be considered as an expression of developmental impairment. Consequently, an individual with a disturbed identity is likely to exhibit more psychological vulnerabilities. For example, an individual with identity diffusion would likely present with poor impulse control, a lack of consistent employment or education, instability in relationships, and be vulnerable to antisocial peer influence, which in turn increases the likelihood of adopting and maintaining antisocial attitudes. It is noteworthy that these presenting problems correspond to the main dynamic risk factors/vulnerabilities that are significantly associated with offending behavior.

## Responsivity Factors

The responsivity principle is central to offender rehabilitation in a correctional setting and includes general and specific responsivity (Bonta & Andrews, 2017). The most relevant to this study is specific responsivity, which suggests tailoring interventions to personal characteristics. Addressing these responsivity variables could enhance the effectiveness of treatment targeting criminogenic needs, and conversely, failing to address responsivity factors might diminish the efficacy of such treatment (McCormick et al., 2017).

Mental health variables like mood disorders, anxiety, problems with psychosocial functioning, and trauma are often categorized under the RNR framework as specific responsivity factors (Rice et al., 2023). Given the high prevalence rates of psychopathology among incarcerated offenders (Fazel et al., 2016), a better understanding of the interaction between mental health problems and criminogenic needs would help improve offence-focused interventions (McCormick et al., 2017). Related to this, however, is the possibility that identity diffusion, given its close association with psychopathology, might also impact a young offender’s responsiveness to treatment. For instance, an individual without a coherent and consistent sense of self may struggle to internalize treatment gains and implement positive, prosocial changes in their life. This includes difficulties maintaining employment, leading a more structured lifestyle, forming and sustaining stable and fulfilling relationships, changing antisocial attitudes, and making decisions that help them resist antisocial peers.

Similarly, individual strengths are also considered specific responsivity factors (Finseth et al., 2022; Singh et al., 2014). Strengths for young offenders include, but are not limited to, the following: positive relationships, emotional stability, problem-solving skills, structured prosocial activities, and parent involvement (Noyori-Corbett & Moon, 2010; Shepherd et al., 2018). From a developmental perspective, it is imperative to comprehend how and when offenders abstain from crime and the role their strengths play in desistance (Wanamaker et al., 2018). While it is still unclear whether the inclusion of protective factors in risk assessment instruments adds any value to predictive accuracy (Dickens & O’Shea, 2017), many researchers have highlighted the important role strengths play in the development of treatment and risk management plans, potentially preventing recidivism at a young age (Shepherd et al., 2018; Wanamaker et al., 2018). One theoretical model, the compensatory model, emphasizes the risk-reducing effect of protective factors (de Vries Robbé, 2014; Serin et al., 2016). It is noteworthy that in criminal justice research, the concept of strengths has been utilized in various capacities, leading to inconsistencies in labeling, conceptualization, and measurement (Wanamaker et al., 2018). In the current research, given that there is no universally agreed-upon definition of protective factors (Viljoen et al., 2020), the terms “strength” and “protective factor” will be used interchangeably.

## Current Research

Identity formation is one of the key developmental milestones of emerging adulthood, and evolves along personal, social, and cultural dimensions, rather than being solely influenced by physiological changes. It is likely that young adult offenders struggle to successfully achieve this developmental task due to a range of antisocial experiences, mental health issues, and further adversity during incarceration. While advocates for a developmental approach suggest tailoring risk assessments as well as rehabilitation programs to meet developmental needs of emerging adults (Farrington et al., 2017; Viljoen, Cruise, et al., 2012), there is little research exploring the relationship between identity formation and dynamic risk factors.

Based on Beech and Ward’s (2004) etiological model, identity formation can be conceptualized as a developmental factor, where identity diffusion represents developmental adversity and contributes to the development of psychological vulnerabilities associated with offending behavior. Furthermore, in the context of the Risk-Need-Responsivity (RNR) model, identity formation, as well as psychopathology and strengths, would typically be viewed as responsivity factors, which would have an impact on responsiveness to correctional treatment. This study aims to examine (1) the relationship between dynamic risk factors, static risk factors, strengths, psychopathology, and identity diffusion, and (2) the value of identity formation in explaining dynamic risk factors in addition to other important factors mentioned above.

Therefore, the following research questions are formulated:

1. What are the associations between criminal history, vulnerabilities, strengths, psychopathology, and identity formation?
2. Can identity formation explain vulnerabilities above and beyond criminal history, strengths, and psychopathology?

## Method

### Participants

The study involved 99 males between ages of 18 and 23 (*M*_age_ = 20.74, *SD* = 1.68), serving custodial sentences in four Lithuanian prisons. Most of the sample were of Lithuanian nationality (91.9%; one case was unknown). Regarding education, the research participants fell into the following categories: those who completed grade 8 (i.e., junior high school) (5.2%); those who completed grade 9 or 10 (66%); and those who completed grade 12 (i.e., graduated from secondary school) or vocational training (28.9%). Index offenses encompassed a variety of crimes, including violent acts such as assault or homicide (30.3%, *n* = 30), property offenses such as theft or robbery (42.2%, *n* = 42); sexual offenses (10.1%, *n* = 10), and drug trafficking (17.2%, *n* = 17).

### Measures

#### START: AV

The Short-term Assessment of Risk and Treatability: Adolescent version (START: AV; Viljoen et al., 2014) was used to measure dynamic risk factors and protective factors. It is a structured professional judgment scheme that guides the assessment of risk of multiple adverse outcomes in adolescents by evaluating items as both vulnerabilities and strengths. A Lithuanian version of the START: AV (Viljoen et al., 2014/2018) was utilized in this study. The START: AV consists of 27 items (e.g., *Coping, Impulse Control, Attitudes, Relationship with Peers* and/or *Caregivers*). Each item was rated on a 3-point scale (0 = *low*, 1 = *moderate*, 2 = *high*), indicating the level of strengths and vulnerabilities observed during the past 3 months. In practice, the scores of structured professional judgment tools are typically not summed (Kleeven et al., 2023). However, for the purpose of the current study, total scores for both the Strengths and Vulnerabilities sections of the START: AV were calculated. Items with few ratings (e.g., Item 23 “Medical Adherence” and Item 25 “Case Specific Items”) were excluded from the analysis, resulting in a score in the range between 0 and 50 for the Strengths and Vulnerabilities scales respectively. In the present sample, the psychometric characteristics of the START:AV were deemed appropriate and are detailed in Table 1.

**Table 1.** Descriptive Statistics of the Variables.

Variable	*M*	*SD*	Range	α	Skewness	Kurtosis
No. sentencing occasions	4.42	3.30	1–15		0.87	0.09
Length of sentence (months)	37.17	27.20	4–123		1.48	1.87
Time already served in prison (months)	14.53	15.01	0–74		1.52	2.20
START:AV Vulnerabilities	18.73	9.42	3–41	.89	0.37	−0.68
START:AV strengths	13.86	6.95	1–34	0.87	0.48	0.43
AIDA	68.29	35.87	2–155	0.94	0.38	−0.56
ASR	48.63	33.50	2–174	0.94	1.38	2.62

*Note.* START: AV = Short-term Assessment of Risk and Treatability: Adolescent Version; AIDA = assessment of identity development in adolescence; ASR = adult self-report.

#### AIDA

The Assessment of Identity Development in Adolescence (AIDA; Goth & Schmeck, 2018) was used to measure identity diffusion. It is a self-report inventory designed to assess identity development in terms of impairments in personality functioning. The Lithuanian version of AIDA (Ragelienė & Barkauskienė, 2020) was utilized in this study. Although originally developed for individuals aged 12 to 18, the basic AIDA model can be generalized to older samples (19+), indicating excellent psychometric properties and showing no remarkable differences in population norms (Sharp et al., 2023). The AIDA consists of 58 items (e.g., *Sometimes I feel like a fake, because my internal thoughts and feelings don’t match my behavior; I am confused about what kind of person I really am*), each rated on a 5-point Likert scale ranging from 0 = *no*, 1 = *more no*, 2 = *part/part*, 3 = *more yes*, to 4 = *yes*. Responses to the AIDA items were summed up for a total score of Identity Diffusion ranging from 0 to 232.

#### ASR

The Adult self-report (ASR; Achenbach & Rescorla, 2003) was used to measure psychopathology. It is a part of the Achenbach System of Empirically Based Assessment (ASEBA) taxonomy. The Lithuanian version of the ASR (Šimulionienė et al., 2016) was used for this study. It consists of 120 items reflecting problems in functioning over the previous 6 months (e.g., *My mood swing between elation and depression; I refuse to talk; I scream or yell a lot*). Each item was rated on a scale ranging from 0 = *not true*, through 1 = *somewhat true*, to 2 = *very true*. Adding up the scores on all problem items yields a *Total problems* score for adult psychopathology, which ranges from 0 to 240.

**Criminal History**: This was defined by three key variables: the number of sentencing occasions, the length of the sentence (in months), and the time already served in prison (in months). The **number of sentencing occasions** refers to how many times an individual has been formally sentenced for a crime. This includes instances of multiple convictions during a single trial, as well as single offenses resulting in sentencing at different times. The **length of the sentence** indicates the total duration of the current imprisonment term. **Time already served in prison** reflects the actual duration that a person has spent incarcerated during their current sentence, without any breaks or releases from prison.

### Procedure

#### Sample Selection

In total, 153 adult males aged 18 to 23 were serving their custodial sentences in four different Lithuanian prisons (*n* = 49, *n* = 21, *n* = 20, and *n* = 62, respectively) at the time of the study. All of them were referred, but 75% (*n* = 115) of those convicted males were present in prisons during visits from March to May 2023 and were invited to participate in the study. Off those invited, 99 young adults (86.1 %) consented to take part in the study and completed the self-reported questionnaires, thus forming the study’s sample. A comparison of sociodemographic characteristics between participants and non-participants showed that the latter group was notably older (Mann Whitney *U* = 531.500, *p* = .032). No differences were found between the two groups in terms of the number of convictions, type of crime, duration of imprisonment sentence or the duration of time already served. Out of the 99 study participants, 79 young offenders attended the START: AV interview.

#### Data Collection

Ethical approval for the study was granted by the Psychological Research Ethics Committee at Vilnius University, Lithuania. Participants were enrolled in the study after providing written, informed consent. They were informed about their right to withdraw from the study at any moment without facing any consequences. The lead author of this article, who has extensive experience in research and conducting risk assessments, was responsible for the data collection and the risk assessment measure scoring. First, study participants were requested to fill in the ASR and AIDA questionnaires and return them in sealed envelopes. Semi-structured interviews were conducted (interview length *M* = 39.53 min, *SD* = 9.42) and the audio-recorded material was used for scoring the START: AV Strengths and Vulnerabilities. The criminal history data was obtained from the case files.

#### Missing Data

Due to limited information, some START: AV Vulnerabilities and Strengths could not be assessed. Following previous research (Viljoen, Beneteau, et al., 2012), a 20% cut-off for missing items (up to five) was applied. In this study, one case had one missing item, and another had two. The total scores were prorated using the formula: prorated total score = [(raw total score/50) × number of missing items] + raw total score (Desmarais et al., 2012). Not all participants provided complete scores for the ASR and the AIDA measures. Missing data were filled using mean imputation if the number of omitted items was within acceptable limits (ASR: up to 8 items; AIDA: up to 7 items). About 24% of participants had imputed scores for the ASR, and 23.2% for the AIDA, aligning with other studies (de Vries et al., 2020). Seven protocols of the AIDA and 10 of the ASR were excluded from the analysis as they exceeded the acceptable limits of missing data.

### Statistical Analysis

Data analysis was carried out using IBM SPSS Statistics version 23. Internal consistency of the scales was assessed using Cronbach’s alpha. Means and variances of the variables were calculated, and the normality of variables was assessed in terms of skewness and kurtosis. To address the research questions of this study, Pearson’s correlation coefficient (*r*) was initially used to examine the relationships between the variables. Following this, hierarchical regression analysis was conducted to assess the incremental utility of identity diffusion in predicting dynamic risk factors beyond criminal history, protective factors, and psychopathology. The dependent variable was START: AV Vulnerabilities. In the regression model, the number of sentencing occasions and the START: AV Strengths were entered as predictors at Step 1, the ASR Total score entered at Step 2, and the AIDA Total score entered at Step 3. Diagnostic tests were conducted to assess the final regression model’s adherence to its underlying assumptions, including normality, homoscedasticity, independence, and non-multicollinearity.

## Results

Descriptive statistics for the criminal history, dynamic risk factors, protective factors, identity formation, and psychopathology variables are shown in Table 1. The analysis of skewness and kurtosis indicated no significant deviations from the normal distribution assumption, except for the ASR and the time already served in prison. Internal consistency of the scales was deemed sufficient.

Table 2 presents the results of a correlational analysis between all the variables of this study across the entire sample. A significant association was found between the number of sentencing occasions and the START: AV Vulnerabilities. This is in line with expectations, indicating that a higher involvement in criminal activities was associated with more dynamic risk factors. The other two criminal history variables – the length of sentence and the time already served in prison - did not demonstrate statistically significant correlations with the START: AV Vulnerabilities, and therefore, they were excluded from further analysis.

**Table 2.** Correlations Between All the Variables.

Variable	1	2	3	4	5	6
1. No. of sentencing occasions						
2. Length of sentence (months)	−.14					
3. Time already served in prison (months)	−.07	.66**				
4. START: AV Vulnerabilities	.23*	.01	.06			
5. START: AV strengths	−.13	.16	.07	−.65**		
6. AIDA	.08	.22*	.14	.43**	−.20	
7. ASR	.04	.24*	.30**	.33**	−.06	.65**

*Note.* Significant correlations bolded. START: AV = short-term assessment of risk and treatability: adolescent version; AIDA = assessment of identity development in adolescence; ASR = adult self-report.

* *p* < .05. **p < .01.

The length of the sentence was found to be positively associated with the ASR and the AIDA. Although these correlations were of small effect sizes (i.e., *r* < .30), they were statistically significant. Furthermore, a significant association was found between the time served in prison and the ASR, suggesting that greater difficulties in psychosocial functioning were experienced with the increased duration of imprisonment.

Ratings of the START: AV Vulnerabilities and Strengths were highly intercorrelated (*r* = −.65). The START: AV Vulnerabilities showed significant positive correlations with the AIDA (*r* = .43) and the ASR (*r* = .33). Also, a high intercorrelation was found between the AIDA and the ASR (*r* = .65).

To examine the factors explaining the dynamic risk factors of young convicted adults, hierarchical regression analysis was conducted with the START: AV Vulnerabilities as the dependent variable. The necessary assumptions for the regression model were tested and met. The data showed no issues with multicollinearity. Additionally, tests confirmed that the residuals were normally distributed, and the assumptions of linearity, homoscedasticity, and independence of residuals were satisfied. The results of the hierarchical regression are presented in Table 3.

**Table 3.** Hierarchical Regression Predicting the Dynamic Risk Factors.

Predictor	β	*t*	*p*	95% CI	*R* ^2^	Δ*R*^2^
Model (Step) 1^a^					.40**	
No. of sentencing occasions	.15	1.55	.126	[**−**0.12 to 0.96]		
START: AV strengths	**−.60**	**−6.21**	**.000**	**[−1.06, −0.55]**		
Model (Step) 2^b^					.48**	.08*
# Sentencing occasions	.16	1.72	.090	[−0.07, 0.95]		
START: AV strengths	**–.61**	**–6.69**	**.000**	**[−1.06, −0.57]**		
ASR	**.28**	**3.08**	**.003**	**[0.03, 0.13]**		
Model (Step) 3^c^					.51**	.03*
No. of sentencing occasions	.15	1.71	.092	[−0.07, 0.92]		
START: AV strengths	**−.56**	**−6.11**	**.000**	**[−1.00, −0.51]**		
ASR	.09	.67	.505	[−0.05, 0.10]		
AIDA	**.26**	**2.02**	**.048**	**[0.00, 0.13]**		

*Note.* Significant results bolded. START: AV = short-term assessment of risk and treatability: adolescent version; ASR = adult self-report; AIDA = assessment of identity development in adolescence.

a Model significant, *F*(2, 66) = 21.90, *p* < .001.

b Model significant, *F*(3, 65) = 19.62, *p* < .001.

c Model significant, *F*(4, 64) = 16.43, *p* < .001.

* *p* < .05. **p < .01.

In the first model, the START: AV Strengths contributed significantly to the regression model, whereas the number of sentencing occasions was not found to be a significant predictor of the dynamic risk factors among incarcerated emerging adults. Together, the two variables accounted for about 40 % of the variance in the START: AV Vulnerabilities. In the second step, the addition of psychopathology as one more predictor to the regression model explained a significant further 8 % of the variance in the dependent variable. Both the START: AV Strengths and the ASR significantly predicted the START: AV Vulnerabilities (β = −.61, *p* = .000, and β = .28, *p* = .003 respectively). In the third step, the AIDA was found to be a significant predictor over and above the variables entered into the model previously (β = .26, *p* = .048). After adding identity diffusion to the model, psychopathology ceased to be a significant predictor of dynamic risk factors. Together, the four independent variables accounted for about 51 % of the variance in the START: AV Vulnerabilities, and the Δ*R*^2^ was significant (*p* = .048).

## Discussion

The study demonstrated that difficulties in identity formation were important in understanding dynamic risk factors among the sample of incarcerated emerging adults. Individuals with higher identity diffusion were found to serve longer sentences and had more mental health issues. Furthermore, identity diffusion, as one of the developmental adversities, was found to explain dynamic risk factors or vulnerabilities above and beyond criminal history, strengths, and psychopathology. This suggests that identity diffusion can be considered as one of the developmental factors significantly informing vulnerabilities, such as poor impulse control, deficits in emotional regulation skills, a lack of consistent employment or education, instability in relationships, susceptibility to the influence of antisocial peers, etc. Similarly, one study found that identity diffusion informed risk factors such as a lack of impulse control and emotional regulation, potentially resulting in reactive aggression (Dammann et al., 2011). The findings are also consistent with the proposition that dynamic risk factors should be conceptualized more as outcomes of underlying psychological issues rather than causes of offending behavior (Ward, 2016), and that these dynamic risk factors might stem from developmental adversities (Beech & Ward, 2004). Based on this, the present study’s authors speculate that addressing identity diffusion in correctional treatment of emerging adults may have the potential to reduce certain criminogenic needs or risk factors, such as poor impulse control, susceptibility to antisocial peer influence, etc. Also, it encourages a consideration of therapy approaches and interventions that are specifically designed to address profound personality-related issues. However, further research is needed to better understand the relationships between identity diffusion, dynamic risk factors, and the risk of offending, as this was beyond the scope of the current study.

Furthermore, the findings of this study add to the body of existing research that promotes an overall developmentally sensitive approach to correctional interventions, risk assessment, and processing of young adults in the criminal justice system (Cauffman et al., 2015; Farrington et al., 2017). In terms of practical implications, the findings suggest that correctional rehabilitation targeting identity development could be particularly effective for incarcerated emerging adults. Incorporating strategies that promote self-reflection and identity exploration may enhance offenders’ responsiveness to treatment, improving rehabilitation outcomes and reducing the risk of re-offending. Moreover, addressing identity-related issues could also help re-engage individuals who had previously withdrawn from correctional rehabilitation, providing a developmentally appropriate framework to foster behavioral change among this group of offenders.

In the present study, criminal history was found to correlate with the dynamic risk factors: a higher number of previous sentencing occasions was associated with more vulnerabilities among young adults. This is consistent with previous research, indicating that individuals with an extensive criminal history are at a higher risk of re-offending in the future (Fries et al., 2013; Kurlychek et al., 2006), and that the increased likelihood of recidivism is often associated with the presence of more dynamic risk factors. Douglas and Skeem (2006) explained the interplay between static and dynamic risk factors, suggesting that the former reflected the inter-individual variability in risk, whereas the latter explained intra-individual variability in criminal potential. However, within the regression model of the current study, a significant relationship between the number of sentencing occasions and dynamic risk factors was no longer observed, likely due to statistical suppression. In the sample of the present study, young adults accumulated numerous sentences for minor offenses alongside lengthy imprisonments for index offenses, and this most likely reflected the tough-on-crime approach still prevalent in the Lithuanian criminal justice system (Dünkel & Sakalauskas, 2017; Sakalauskas et al., 2020).

The findings of this study confirmed a relationship between incarceration and the mental health of emerging adults. Longer time spent in prison was found to be associated with more self-reported psychopathology. These results were consistent with research suggesting a detrimental impact of imprisonment on young offenders’ psychological well-being (Cunha et al., 2023). However, identity formation had additional value over and above psychopathology in explaining dynamic risk factors among young offenders. While psychopathology undoubtedly plays a role in shaping behavior, the disruption in identity development may have a more profound impact on an individual’s development trajectory.

Regarding the ongoing discussion in the scholarly literature about the interplay between risk and protective factors (Polaschek, 2016), the current study supports a risk-reduction model, which posits that protective factors have a diminishing effect on dynamic risk factors (de Vries Robbé, 2014). This finding has significant practical implications, suggesting that interventions aimed at enhancing protective factors may contribute to reducing vulnerabilities and mitigating the risk of re-offending. In the criminal justice context, this entails improving treatment outcomes by identifying, addressing, and building on young offenders’ strengths. The ultimate purpose of this approach is not only to reduce the risk of re-offending but also to empower young offenders to become productive, purposeful, and valuable members of society (Serin et al., 2016). These results also align with the growing recognition that a holistic, strengths-based approach to correctional rehabilitation can offer more sustainable and meaningful outcomes than solely focusing on risk management.

The present study did not provide evidence for a notable association (*r* = −.20, n.s.) between Identity Diffusion and the START: AV Strengths, most probably because of the relatively small sample size. Other studies found that strengths, like closer relationships with significant others and the support received from them, were related to the development of a coherent and integrated sense of self (Hasanah et al., 2019). Therefore, further research is needed for a more thorough understanding of the interplay between strengths and the continuum of identity formation.

## Limitations and Future Directions

The inter-rater reliability of the START: AV ratings was not utilized in this study, as the assessment of the START-AV Strengths and Vulnerabilities were conducted by a single assessor. While this approach may have potentially diminished the accuracy of the scores, it is worth noting that the assessor has extensive experience in conducting this type of assessment. In previous studies in which she participated as an assessor, inter-rater variability was found to range from fair to excellent (Klimukienė et al., 2018).

Another limitation of the study is the use of a cross-sectional design, as it does not capture the dynamics of the constructs and their interrelations over time. Longitudinal studies would provide more information about the temporal relationships between identity development and dynamic risk factors.

This study exclusively focused on male participants, and thus, the findings cannot be generalized to the female population. Given that females in the population sample tend to score significantly higher than males on the AIDA Identity Diffusion scale (Karvonen et al., 2022), this indeed appears to be an important area for further research. Considering that this study is among the first to explore how developmental issues inform dynamic risk factors among the offender population, there is a clear need for further research involving larger and more diverse populations to validate and expand upon our findings.
